# Cadmium Sulphide-Reduced Graphene Oxide-Modified Photoelectrode-Based Photoelectrochemical Sensing Platform for Copper(II) Ions

**DOI:** 10.1371/journal.pone.0154557

**Published:** 2016-05-13

**Authors:** I Ibrahim, H. N Lim, N. M Huang, A Pandikumar

**Affiliations:** 1 Department of Chemistry, Faculty of Science, Universiti Putra Malaysia, 43400 UPM, Serdang, Selangor, Malaysia; 2 Functional Device Laboratory, Institute of Advanced Technology, Universiti Putra Malaysia, 43400 UPM, Serdang, Selangor, Malaysia; 3 Low Dimensional Materials Research Centre, Department of Physics, Faculty of Science, University of Malaya, Kuala Lumpur, 50603, Malaysia; Institute for Materials Science, GERMANY

## Abstract

A photoelectrochemical (PEC) sensor with excellent sensitivity and detection toward copper (II) ions (Cu^2+^) was developed using a cadmium sulphide-reduced graphene oxide (CdS-rGO) nanocomposite on an indium tin oxide (ITO) surface, with triethanolamine (TEA) used as the sacrificial electron donor. The CdS nanoparticles were initially synthesized *via* the aerosol-assisted chemical vapor deposition (AACVD) method using cadmium acetate and thiourea as the precursors to Cd^2+^ and S^2-^, respectively. Graphene oxide (GO) was then dip-coated onto the CdS electrode and sintered under an argon gas flow (50 mL/min) for the reduction process. The nanostructured CdS was adhered securely to the ITO by a continuous network of rGO that also acted as an avenue to intensify the transfer of electrons from the conduction band of CdS. The photoelectrochemical results indicated that the ITO/CdS-rGO photoelectrode could facilitate broad UV-visible light absorption, which would lead to a higher and steady-state photocurrent response in the presence of TEA in 0.1 M KCl. The photocurrent decreased with an increase in the concentration of Cu^2+^ ions. The photoelectrode response for Cu^2+^ ion detection had a linear range of 0.5–120 μM, with a limit of detection (LoD) of 16 nM. The proposed PEC sensor displayed ultra-sensitivity and good selectivity toward Cu^2+^ ion detection.

## 1. Introduction

Copper (II) ions (Cu^2+^) are the third most abundant transition metal ion and an essential trace element in the human body. Although Cu^2+^ ions are physiologically essential for various reasons, they are also an important environmental pollutant. The recommended allowance of copper is 0.8–0.9 mg/day for normal adults [1] The drinking water quality guidelines by the World Health Organization (WHO) suggests that the amount of copper in drinking water should be limited to 1.3 mg/L (∼20 μM) [2, 3] Aberrant levels of copper exhibit significant deleterious effects on living organisms, such as Wilson’s disease, pre-menstrual syndrome, adult polycystic kidney disease, fibroids, and infertility [3, 4] As a result, there has been ongoing interest in developing better sensors for Cu^2+^ ion detection, because of their significance in the environment and biological systems. Although conventional methods such as chromatography-mass spectrometry, inductively coupled plasma mass spectrometry, and atomic absorption spectrometry are common detection tools, expensive instrumentation, extremely time-consuming processes, and the necessity of sophisticated operation have retarded their progress. Hence, the discoveries of sustainable techniques are needed to overcome the limitations. Recently, photoelectrochemical (PEC) has attract much attention among researcher as a sensing platforms for Cu^2+^ ion detection because its offered sensitive, fast, reproducible, simple, low-cost, and accurate potential alternatives to the above conventional methods.

PEC sensing is an approach that relies on photo-illumination to transfer electrons between an analyte, a semiconductor, and an electrode [5, 6] A PEC sensor harnesses light to stimulate the photoactive species on the electrode, and utilizes a photocurrent as a means to quantify the changes. Furthermore, it has numerous inherent benefits such as a simple electronic circuit and easy fabrication, and it can use a low-power light source, which results in a very low instrumentation cost [7] In order to further enhance the performance of PEC sensors, triethanolamine, ascorbic acid, and sodium sulphide are often added to the PEC detection cell as electron donors [8] Semiconductors such as TiO_2_ [9] ZnO [10] and CdS [11] can be used as the photoactive materials in the PEC sensor.

The photoactive material plays a major role in the PEC sensing performances for heavy metal ions. A PEC sensing system with high sensitivity can be achieved by amplifying the photocurrent intensity and minimizing the electron−hole recombination. Cadmium sulfide (CdS) semiconductor nanoparticles are the best candidate to achieve this target because of their application potential, based on size-tunable optical properties and high mobility [12] CdS possesses a band gap of ~2.4 eV, and has been considerably investigated for PEC sensing, as well as for the other visible-light-driven PEC applications [13] In order to heighten the photocurrent generated by the CdS nanoparticles, it is essential to hold back the rapid recombination of the photogenerated electron–holes using CdS on support materials with large surface areas or by hybridizing CdS with conductive matrices such as carbon materials [14, 15]

As a flexible atom-thin 2D carbon material, graphene has emerged as a rising star because of its outstanding properties and excellent performance, including its very large surface area (theoretically, 2630 m^2^ g^−1^ for a single-layer of graphene) [16], extraordinary electrical and thermal conductivities [17, 18], excellent mechanical strength [19] and good biocompatibility [20]. Considering its remarkable physical, chemical, and structural properties, materials based on graphene and its derivatives have sprung up quickly and been given tremendous attention across many disciplines, including novel nanoelectronics, high-frequency electronics, energy conversion and storage, biomedical devices, and sensors. Graphene-based electrochemical studies have received much interest because of its important role in accelerating electron transfer, with the merits of a high carrier mobility and exceptional electrical conductivity [21], which subsequently provide new opportunities to develop high-performance electrochemical sensors. To date, most graphene-based electrochemical sensors have used reduced graphene oxide (rGO) instead of graphene oxide (GO) because (i) it is rich in structural defects and its chemical groups facilitate the charge transfer process and thus enhance its electrochemical activity; (ii) the abundant chemical moieties on the rGO surface create availability and flexibility for various surface functionalizations, which boost the sensing performance; (iii) excellent tunable chemical and electrical properties; and (iv) efficient charge transport compared to non-conductive GO. The aforementioned benefits of rGO in electrochemical sensing can be enhanced by assembling CdS nanoparticles on its surface to intensify the photocurrent performance through the synergic effects between CdS and rGO [21] in addition to addressing the photocorrosion problems of CdS nanoparticles, making this nanocomposite suitable for photoelectrochemical sensing. The present research focused on the development of an easy-to-use, economical, mobile, selective, and sensitive analytical approach for the detection of Cu^2+^ ions based on a photoelectrochemical technique. CdS semiconductor nanoparticles were synthesized on an indium tin oxide (ITO) electrode with an aerosol-assisted chemical vapor deposition (AACVD) procedure using Cd(CH_3_COO)_2_·2H_2_O and CH_4_N_2_S as the precursors to Cd^2+^ and S^2-^, respectively. The rGO was then assembled on the CdS nanoparticles using a dip-coating method. The as-prepared ITO/CdS-rGO electrode was used for the detection of Cu^2+^ ions in an aqueous solution, in the presence of an electron donor, triethanolamine (TEA). It was found that the nanocomposites enhanced the photocurrent response up to 63 μA as a result of the rapid interfacial electron-transfer process. The results of this study demonstrated that the ITO/CdS-rGO photoelectrode has the potential to exhibit an excellent photocurrent response in 0.1 M of KCl under light illumination, which could provide a remarkable response upon the addition of Cu^2+^ ions. Based on the significant quenching in the photocurrent signal, an unconventional strategy was developed for the PEC detection of Cu^2+^ ions, with a linear concentration range of 0.5–120 μM, which gives a LoD of 16 nM.

## 2. Experimental Methods

### 2.1. Materials

Graphite flakes were purchased from Asbury Graphite Mills Inc., USA. Cadmium acetate dihydrate (Cd(CH_3_COO)_2_·2H_2_O) and copper(II) sulfate pentahydrate (CuSO_4_.5H_2_O) were purchased from Hamburg Chemical. Thiourea (CH_4_N_2_S) was purchased from Fisher scientific. Methanol (CH_3_OH, 99.9%) and acetone ((CH_3_)_2_CO, 99.5%) were purchased from Friendemann Schmidt. Ethanol (CH_3_CH_2_OH_2_, 95%) was purchased from Systerm. Potassium chloride (KCl) was purchased from Merck. Triethanolamine (TEA, 99%) was received from R & M Chemicals. Indium tin oxide (ITO) conducting glass slides (7 Ωsq^-1^) were commercially supplied by Xin Yan Technology Limited, China. Stock solutions of Cu^2+^, Ni^2+^, Co^2+^, Fe^3+^, Mn^2+^, Na^+^, K^+^, Ba^2+^, Zn^2+^, Mg^2+^, and Al^3+^ were prepared by dissolving suitable amounts of the CuSO_4_, NiSO_4_, CoSO_4_, Fe(NO_3_)_3_, MnCl_2_, NaCl, KCl, BaCl_2_, ZnSO_4_, MgCl_2_, and AlCl_3_ compounds, respectively, in Milli-Q water with a resistivity larger than 18 MΩ cm. Each sample solution was 10 mL. The entire experiment employed deionized water. The reagents and materials were used as received without further purification.

### 2.2. Fabrication of CdS thin film by AACVD method

A CdS thin film was prepared on an ITO glass substrate (1 cm × 1.5 cm) using an in-house AACVD assembly process. The synthesis strategy was reported earlier by our group [22, 23] The ITO glass was ultrasonically cleaned in acetone, ethanol, and deionized water before being used. Then, the CdS films were prepared from 0.05 M Cd(CH_3_COO)_2_·2H_2_O and 0.1 M CH_4_N_2_S in 35 mL of a methanol solution that was generated into an aerosol to be deposited as CdS on the ITO in the reaction chamber.

### 2.3. Fabrication of photoelectrochemical sensor electrode

The deposition of 0.1 M GO, which was produced using a simplified Hummer's method [24] on the surface of the AACVD-prepared CdS thin film was carried out using a dip-coating method, as reported by our group [22]. The CdS-GO thin film was placed in a furnace at 150°C, and argon gas was flowed for 15 min at a flow rate of 50 mL/min for the reduction process. The electrode was labeled as CdS-rGO.

### 2.4. Characterization techniques

The surface morphologies of the nanocomposites were analyzed using a field emission scanning electron microscope (FESEM, FEI Quanta SEM Model 400 F) equipped with an energy dispersive X-ray (EDX) accessory. The crystalline phase of the CdS-rGO nanocomposite was studied using a Philips X’pert system X-ray powder diffractometer with Cu Kα radiation (λ = 1.5418 Å), and a Raman spectral analysis was carried out using a Renishaw inVia Raman microscope with laser excitation at λ = 514 nm.

### 2.5. Photoelectrochemical studies

Photoelectrochemical measurements were performed using a homemade photoelectrochemical system containing a 150-W halogen lamp (Halloid) as the illumination source. The photocurrent signal was measured on a computer-controlled Versa-STAT-3 electrochemical analyzer from Princeton Applied Research. A CdS-rGO-modified photoelectrode with an active area of 1 cm^2^ was used as the working electrode, and a Pt wire and saturated Ag/AgCl were used as the counter and reference electrodes, respectively. The working electrode potentials stated in this paper are with reference to the Ag/AgCl, unless otherwise stated. All the photocurrent measurements were conducted by dipping the CdS-rGO-modified photoelectrode into a mixture of 0.1 M KCl and 0.5 M TEA at a constant potential of 0 V vs. Ag/AgCl.

## 3. Results and Discussion

### 3.1. Morphological studies

**Fig 1:**
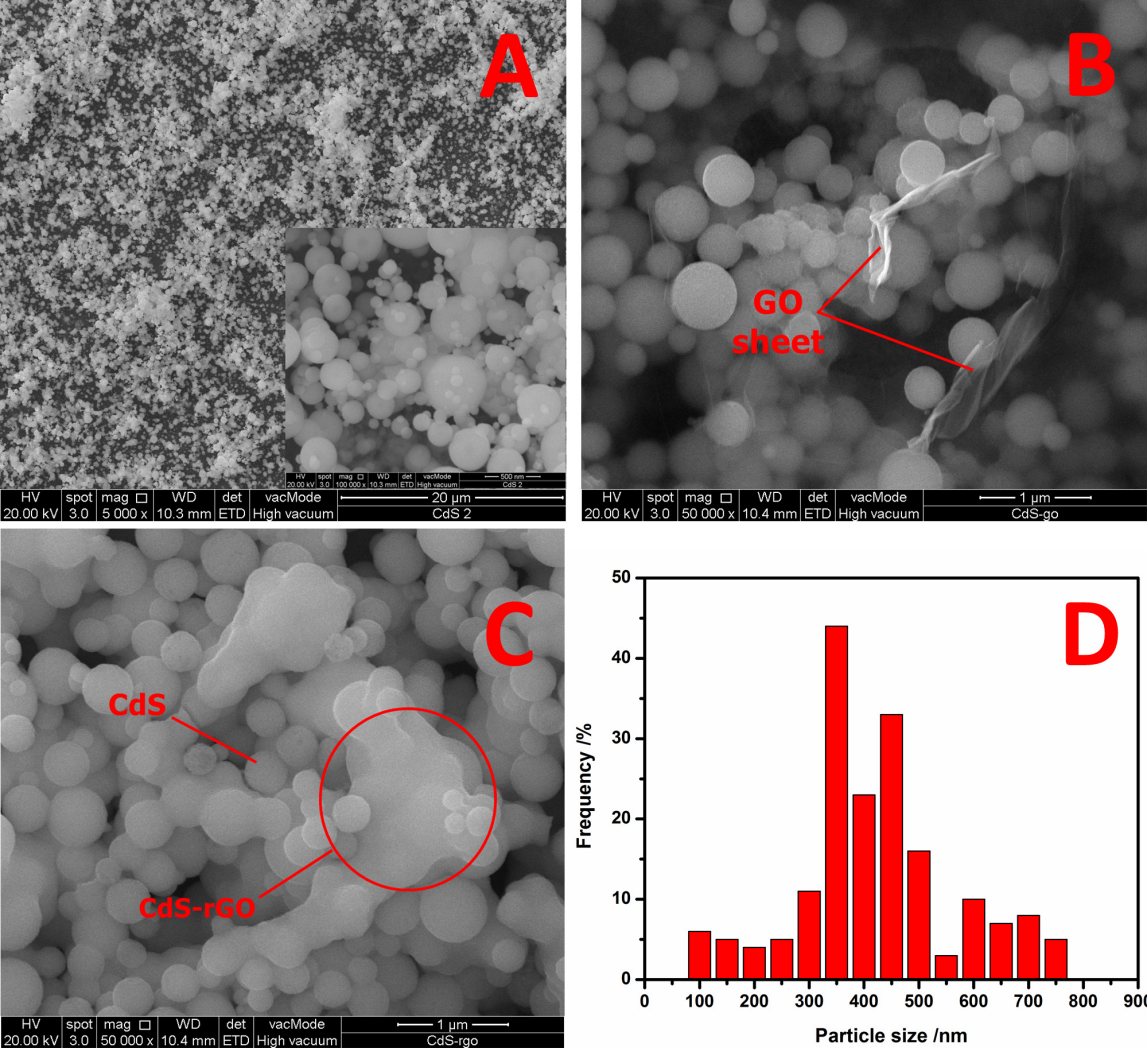
FESEM images of (A) CdS nanospheres, (B) CdS-GO, and (C) CdS-rGO, as well as (D) histogram showing size distribution of CdS nanoparticles in CdS-rGO nanocomposite.

**Fig 1(A)** shows a low-magnification FESEM image of the CdS obtained using the AACVD method at 400°C for 180 min. This image indicates that the sphere-like morphology of pure CdS was composed of homogenous nanospheres with a narrow diameter distribution of about 350–400 nm. These nanospheres were dispersed with good monodispersity (**Fig 1(A), inset**), although a few seemed to agglomerate. Upon the addition of 0.1 mg/ml GO, a transparent ultrathin GO layer was clearly observed on the surface of the CdS nanoparticles (**Fig 1(B)**). The GO nanosheets were curled and corrugated, and CdS nanospheres were distributed over the GO sheets. The GO was then reduced by sintering at a temperature and pressure of 150°C and 50 mL/min, respectively. **Fig 1(C)** shows that the rGO formed a continuous network with CdS nanoparticles, resulting in excellent hybridization of the rGO and CdS. The rGO sheets blanketed the CdS nanoparticles, which enhanced the photo-generated carriers separation, and consequently elevated the PEC performance [25]. The corresponding histograms of the particle size distribution (**Fig 1(D)**) are also shown, along with the FESEM images. The size distribution was determined based on the measurement of 180 CdS nanospheres. It is worth noting that aggregates consisting of small particles were not taken into account in the determination of the particle crystal size. It was found that most of the particles had sizes of <400 nm, and the average particle size was 350–400 nm, as indicated in the histogram.

### 3.2. X-ray diffraction analysis

**Fig 2:**
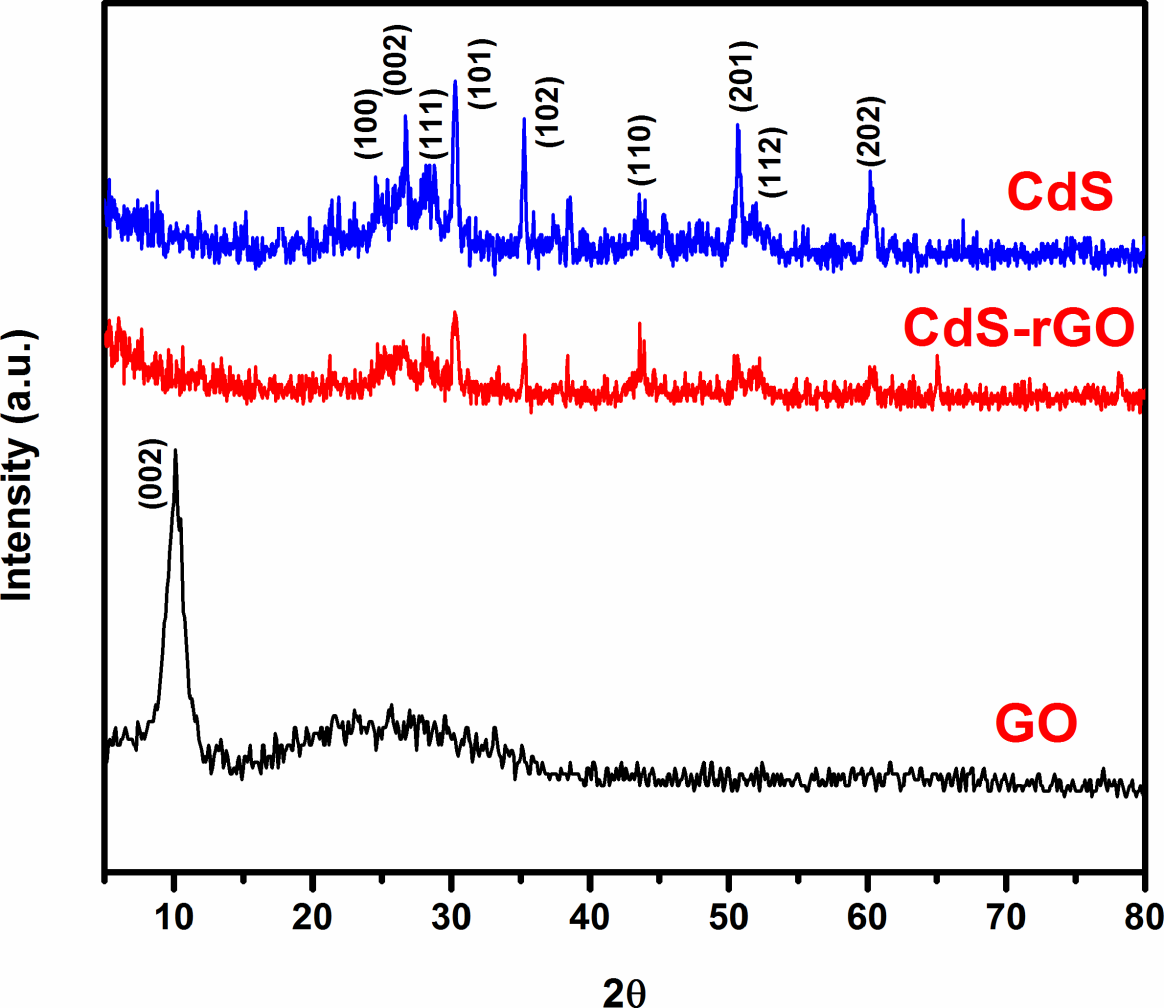
XRD patterns of as-prepared GO, CdS, and CdS-rGO samples.

XRD patterns revealed the effect of the graphene on the crystallinity of the CdS nanoparticles for the GO, CdS, and CdS-rGO films. Their diffraction patterns are given in **Fig 2.** The XRD pattern of GO portrays a distinguishable peak at 2θ = 10.6° at the index of (002). The characteristic peaks at 245°, 26.7°, 26.8°, 30.3°, 35.2°, 43.5°, 50.6°, 51.9°, and 60.2° respectively correspond to the (100), (002), (111), (101) (102), (110), (201), (112), and (202) planes of the hexagonal crystal structure of CdS (JCPDS card no.: 02–0549). The XRD pattern of the CdS-rGO nanocomposite only shows the peaks for the hexagonal CdS phase. However, the nanocomposite shows a peak with lower intensities than those for pure CdS. This would be caused by the graphene wrapped around the surface of the CdS particles, which could induce crystal diffraction. Moreover, no obvious peak is observed at the (002) index for the CdS-rGO composite, which corresponds to the oxygen-containing functional groups located between the layers of GO, suggesting that the GO was reduced to rGO during the heating treatment, and the interlayer spacing was decreased [26].

### 3.3. Raman spectral analysis

**Fig 3:**
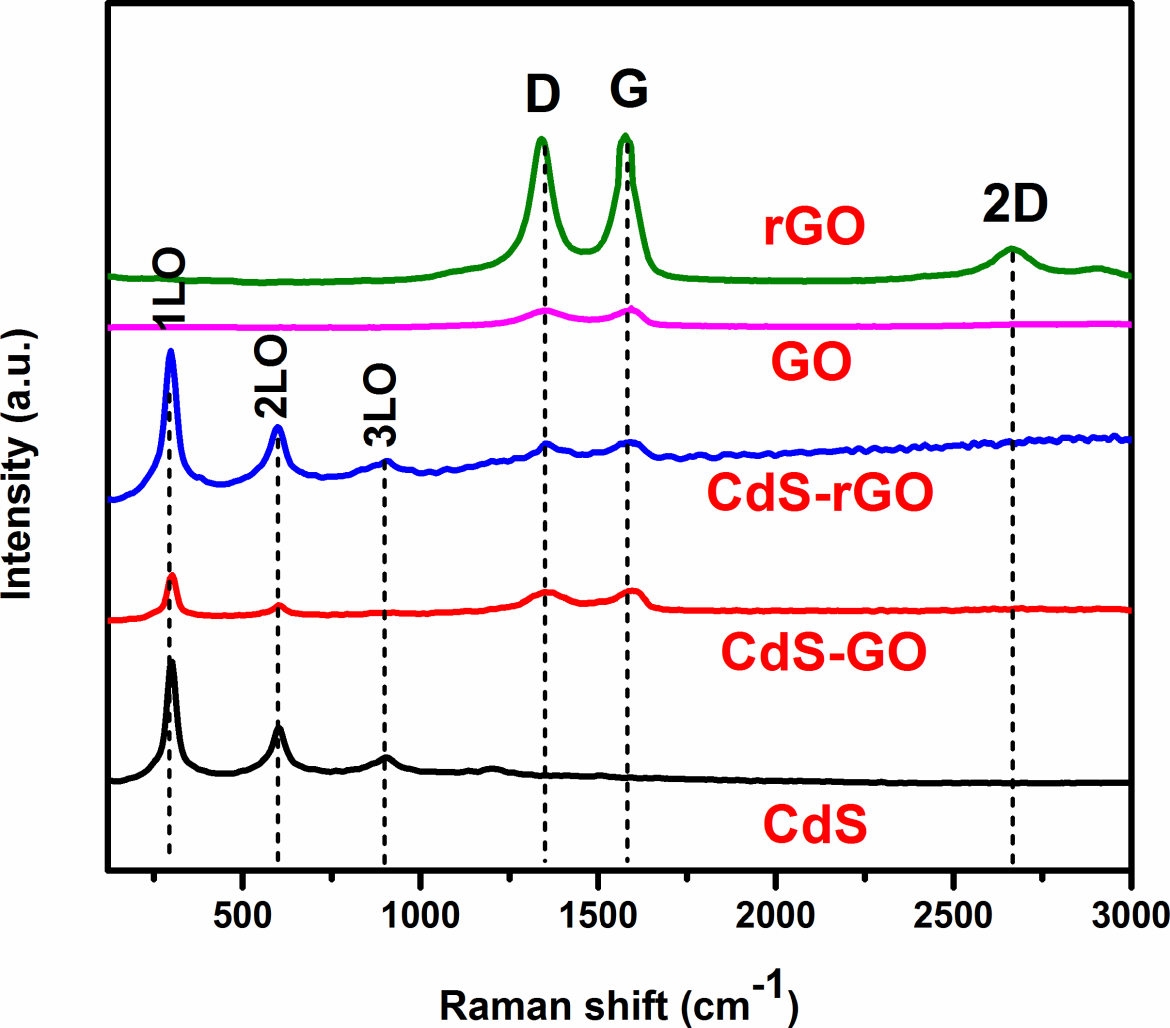
Raman spectra of GO, rGO, CdS, CdS-GO, and CdS-rGO.

Raman spectroscopy was performed on the GO, rGO, CdS, CdS-GO, and CdS-rGO nanocomposites, and the results are shown in **Fig 3.** The Raman profile of GO the typical D and G bands assigned to the қ-point phonons of the A_1g_ symmetry and E_2g_ phonon of the sp^2^ carbon at 1353 and 1593 cm^-1^, respectively. These two bands shifted to lower wavelengths of 1340 and 1577 cm^-1^ after the reduction. The chemical reduction of GO enhanced the intensity ratio of the D to G bands (I_D_/I_G_) from 0.974 to 0.986. The increase in the I_D_/I_G_ ratio for rGO was due to the restoration of the sp^2^ network and the formation of unrepaired defects after the removal of numerous oxygen functionalities [27, 28]. Hence, it gives a clear indication of the effective reduction of GO. Furthermore, the appearance of the 2D band at 2662 cm^-1^ in the rGO spectrum indicates the reduction of GO to rGO [27]. The characteristic peaks of CdS presented at 301, 599, and 905 cm^−1^ correspond to the first-, second-, and third-order longitudinal optical phonon modes (1LO, 2LO, and 3LO), respectively. For the CdS-rGO nanocomposite, all the Raman bands for CdS and rGO can be found. When CdS nanoparticles are deposited on graphene, the intensity of the D band increases relative to the G band, and both bands shift to higher wavenumbers [29]. In the present study, the D and G bands for the CdS/rGO composite appeared at 1355 and 1593 cm^−1^, respectively. The intensity ratios of the D and G bands for CdS–GO and CdS-rGO were 0.989 and 0.991, respectively. The I_D_/I_G_ ratios for these two nanocomposites were higher than those for GO, indicating that a significant number of structural defects were introduced to the graphene lattice in the reaction. Conclusively, the larger I_D_/I_G_ ratios in the composite materials compared to GO indicate an increase in the amount of smaller sp^2^ domains and the revival of graphene network conjugation (re-aromatization) [30]. Moreover, the revived graphene network size was smaller than that of the GO starting material. This effect gave rise to an increased I_D_/I_G_ ratio in the CdS/rGO composite materials [31].

### 3.4. Electrochemical impedance spectroscopy analysis

**Fig 4:**
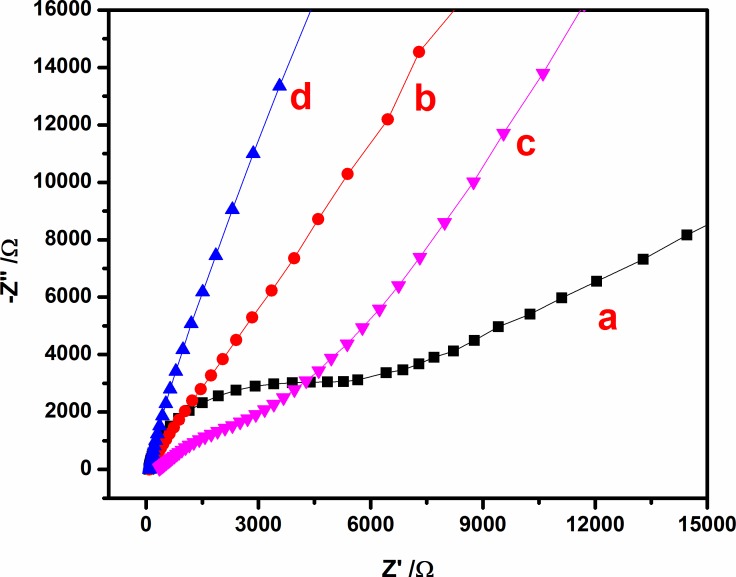
Nyquist plots obtained for (a) CdS, (b) CdS-GO, (c) CdS-rGO, and (d) CdS-rGO-Cu after addition of 4 μM Cu^2+^ ions into 0.1 M KCl and 0.5 M TEA solution.

The electron transfer characteristics and recombination processes at the electrolyte and electrode interfacial surface were investigated using the electrochemical impedance spectra (EIS) in 0.1 M KCl and 0.5 M TEA, as shown by Nyquist plots (**Fig 4**). The EIS were found in a frequency range of 10 kHz to 0.01 Hz to evaluate the frequency response of the nanocomposites. The equivalent series resistance (ESR) was extracted from the x-intercept of the Nyquist plot to evaluate the resistance of the KCl and TEA mixture solution, intrinsic resistance of the nanocomposite, and contact resistance at the interface between the electrode and current collector [32, 33]. The ESR values for the CdS, CdS-GO, CdS-rGO, and CdS-rGO-Cu were 89.14, 88.24, 72.37, and 351.22 Ω, respectively. The CdS-rGO electrode (**Fig 4,** curve c) shows a lower resistance value, indicating that the presence of rGO improved the transfer of the charge performance of the CdS electrode. Meanwhile, the larger resistance value of the CdS-rGO-Cu electrode (curve d) was due to the presence of Cu^2+^ ions on the CdS-rGO electrode, which hindered the electron transfer to the as-prepared electrode, increasing the intrinsic resistance of the nanocomposite. In the high-frequency range, the sizable semi-circle indicates the high charge transfer resistance (R_ct_) caused by the weak transfer of the generated electrons and the recombination properties [10]. In the Nyquist plots, CdS-rGO exhibits a smaller semi-circle of 10.85 Ω compared to CdS-GO (25.33 Ω), suggesting that the electrochemically unstable oxygenous groups were removed during the reduction, hence overcoming the pseudocapacitance [34]. The CdS-GO (curve b) appears to have a large R_ct_ because of excessive oxygenated species, which produced an insulating behavior and obstructed its electrochemical characteristics [35]. Obviously, bare CdS electrodes (curve c) appear to have the largest semi-circle of 8841.2 Ω, particularly because of the absence of rGO, which behaved as an electron transfer medium for CdS excitation [36]. The CdS-rGO-Cu shows a slightly high Rct value of 7700.4 Ω, demonstrating that the Cu^2+^ ions were successfully incorporated on the surface of the sensor electrode [36], which provided a sensitive sensing platform for Cu^2+^ ion detection. Additionally, the low-frequency region portrays a straight line caused by a diffusion-limiting process that is more vertical for CdS-rGO, implying even more diffusion routes for electrons within the CdS-rGO electrode [34], and hence rapid electrolyte diffusion on the electrode–electrolyte interfaces [37].

### 3.5. Photoelectrochemical performance

**S1(A) Fig** shows the linear sweeps of the CdS-rGO electrode in a potential range of -0.6 to 1.0 V versus Ag/AgCl in 0.1 M KCl electrolyte. Under irradiation, the CdS-rGO electrode achieved a maximal current of 100.07 μA at an applied bias of 0.96 V versus Ag/AgCl. Meanwhile, the current was obviously low in the absence of light irradiation, and it had a maximum current of 89.87 μA. The light alternated in 30-s intervals (light on and off) showed the comparable and instantaneous photocurrent response under light and dark conditions due to the PEC effect [38]. Likewise, **S1(B) Fig** appears to have a similar LSV to that in **S1(A) Fig** but the photocurrent response for **S1(B) Fig** is much more pronounced. The current response upon light illumination was 163.03 μA at 0.70 V versus Ag/AgCl, while in the dark condition, it had a current response of 140.47 μA with the same applied bias. The enhanced photocurrent was due to the introduction of 0.5 M TEA in the electrolyte. This is because TEA is a suitable sacrificial electron donor, leading to rapid electron–hole separation, and thus magnifying the photoelectrochemical responses and minimizing the over potential [39]. Conclusively, the CdS-rGO electrode worked extremely well in the presence of TEA, thus giving a stronger current response because more electrons were brought together from the PEC reaction, which suggests an improved separation efficiency [40]. Upon the addition of 4 μM of Cu^2+^ ions, the photocurrent dropped even more dramatically (**S1(C) Fig**). The current was reduced to 67.41 μM under light irradiation and 44.96 μM in the dark condition, both at an applied bias of 0.60 V versus Ag/AgCl. The photocurrent decrease corresponded to the extensive carrier recombination rate [41]. The obvious increase in cathodic current at potentials of -0.20 to -0.10 V was a result of the reduction in Cu^2+^ ions, corresponding to the following reaction: Cu^2+^ + e^-^ → Cu^+^. Further, the influence of the photocurrent performance was investigated in the absence and presence of 4 μM Cu^2+^ ions with 0.1 M KCl and 0.5 M TEA under the light “on-off” condition (**S1(D) Fig**). It was clear for both the absence and presence of Cu^2+^ ions that the current was higher under light illumination and declined when the light was cut off. Upon the addition of Cu^2+^ ions into the solution, there was a reduction in the photocurrent compared to the former value. This suggests that the photocurrent quenching was due to the photocatalytic reduction of Cu^2+^ to Cu^+^ ions. From the results obtained, the photocurrent increased as the electrode potential was made more positive, suggesting that an anodic photocurrent appeared, with electrons as its majority carriers [42]. Thus, the ITO/CdS-rGO electrode behaved as an n-type semiconductor electrode. The n-type material induced oxidation on the electrolyte species at the semiconductor-electrolyte during the PEC performance [42].

**Fig 5:**
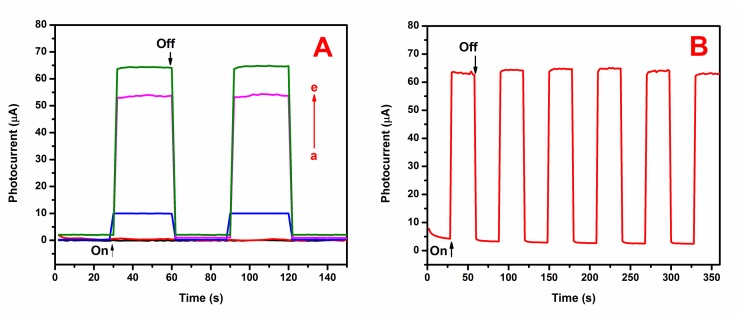
(A) Photocurrent responses of (a) rGO-, (b) GO-, (c) CdS-, (d) CdS-GO-, and (e) CdS-rGO-modified electrodes and (B) time-based photocurrent response of ITO/CdS-rGO in 0.1 M KCl and 0.5 M TEA under chopped irradiation.

Subsequently, the PEC response was investigated by employing different materials to decorate the ITO sheet used as a photoanode. The photocurrent of each prepared sample was calculated in a 0.1 M KCl solution containing 0.1 M TEA under “on-off” light illumination cycles at a bias of 0 V vs. Ag/AgCl, as shown in **Fig 5(A)**. The photoresponse for rGO was non-existent because rGO could not be excited, as shown in curve a [43]. Similarly, for GO (curve b), no obvious photocurrent was observed. This was typically due to the nature of graphene oxide, which is electrically insulating and requires further reduction steps to generate a photocurrent signal [44]. After modification by the AACVD process, the ITO/CdS electrode increased the photocurrent to 9.96 μA (curve c). For the ITO/CdS-rGO electrode, this could boost the photocurrent by at least 6-fold, reaching a value of 64.35 μA (curve e). CdS nanoparticles were bound to the surface of the rGO nanosheets through the van der Waals force [45]. The binding force enabled the CdS nanoparticles to integrate intimately with the ITO electrode. The encapsulation of CdS by the rGO improved the interfacial contact, which facilitated the electron transfer between the CdS and ITO, thus preventing possible current leakage in the ITO/CdS-rGO electrode. Besides, rGO is also capable of dividing and accumulating the photogenerated electrons, which minimizes the recombination rate of the photogenerated electrons and holes, and boosts the photocurrent production [46, 47]. Meanwhile, for the ITO/CdS-GO electrode (curve d), a slightly lower signal (16.27% or 53.88 μA) was revealed compared to CdS-rGO. This was because the electrical conductivity of rGO is higher than that of GO [48].

The stability of the amperometric response of the CdS-rGO-modified photoelectrode in the presence of 0.5 M TEA under the light “on-off” condition is given in **Fig 5(B)**. This graph displays the photocurrent response of the ITO/CdS-rGO based on time, which was repeatedly measured six times at 30-s intervals under visible irradiation. No current was observed in the dark, which clearly suggests that no photoinduced charge separation took place. Interestingly, when the light was turned on, the photocurrent intensity was significantly increased to 64.35 μA. This may have been due to the photoinduced electron–hole separation at the CdS, in which the holes were scavenged by the TEA, and rGO acted as an electron transfer medium. Thus, the electrons were transported to the ITO electrode, resulting in photocurrent generation. The electrode showed a pronounced and stable photocurrent response during the light “on-off” condition, which it maintained. The relative standard deviation was only 1.02%, which implied that the constructed PEC sensor had a stable photocurrent activity.

### 3.6. Photoelectrochemical detection of copper(II) ions

**Fig 6:**
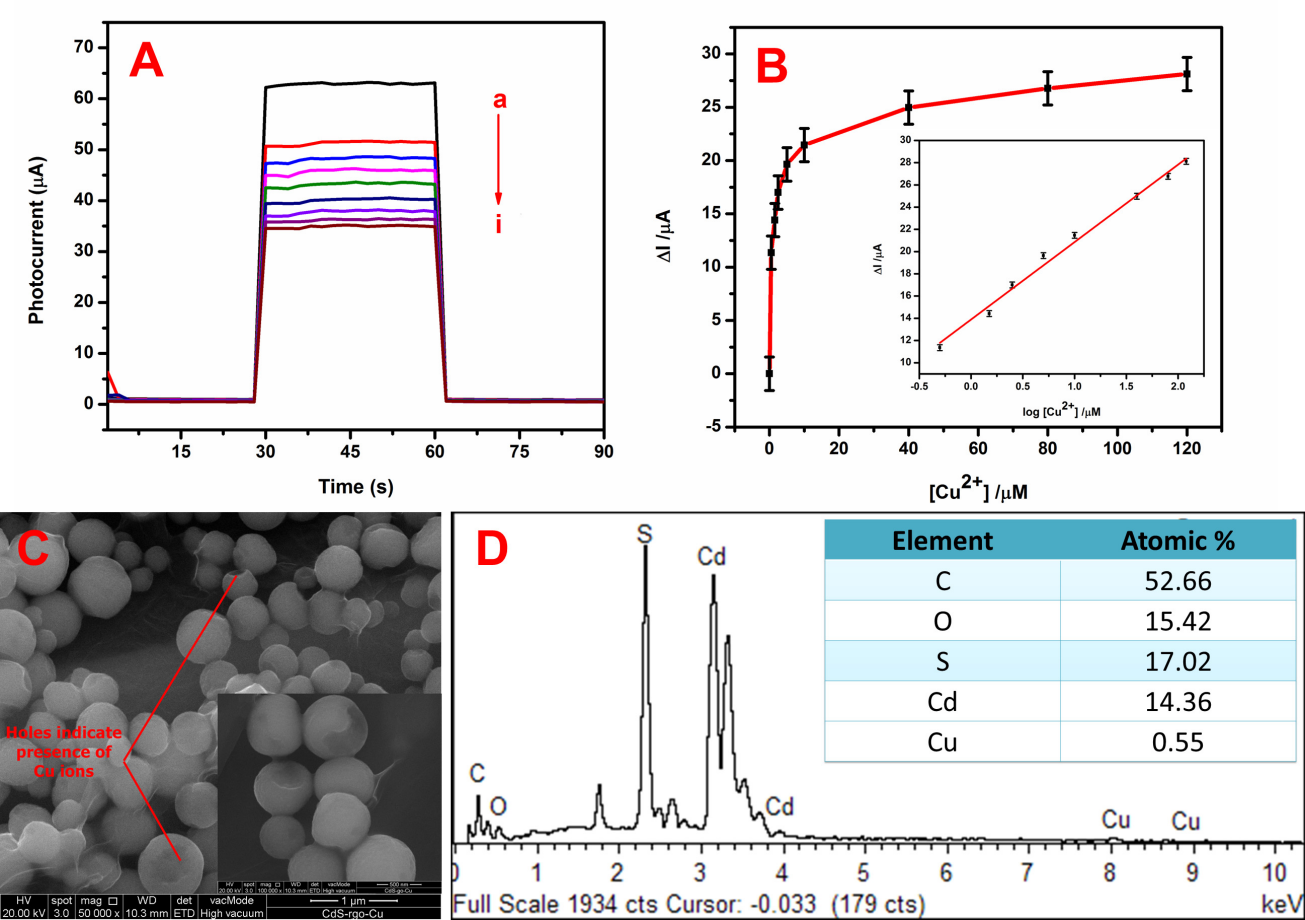
(A) Photocurrent responses of ITO/CdS-rGO electrode at different concentrations of Cu^2+^ ions: (a) 0, (b) 0.5, (c) 1.5, (d) 2.5, (e) 5.0, (f) 10.0 (g) 40.0, (h) 80.0, and (i) 120 μM, and (B) photocurrent change against Cu^2+^ ions concentration. Inset: corresponding calibration curve. (C) Low-magnification image of CdS-rGO-Cu, inset: high-magnification image of CdS-rGO-Cu, and (D) EDX spectrum of CdS-rGO-Cu, inset: weight percentage of elements found in spectrum.

**Fig 7:**
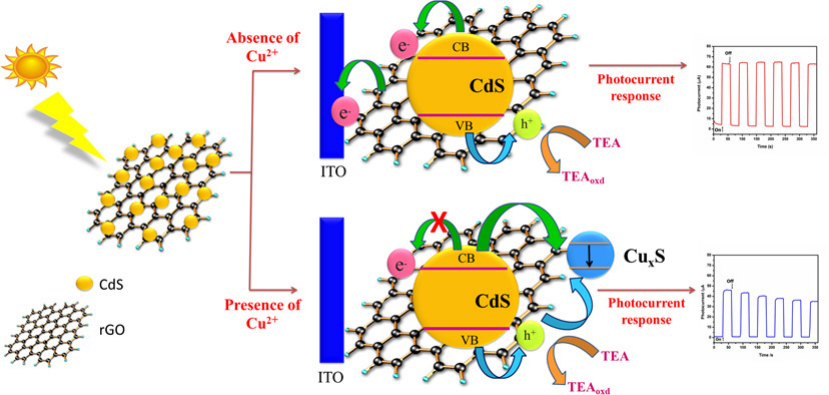
Mechanism for sensing Cu^2+^ ions based on ITO/CdS-rGO nanocomposites.

As shown in **Fig 6(A)**, the PEC performance of the CdS-rGO was evaluated by the sensing of Cu^2+^ ions. The photocurrent density of the ITO/CdS-rGO decreased with increasing concentration of Cu^2+^ ions. **Fig 6(B)** shows the increase in the photocurrent ΔI (ΔI = I − I_0_) and the Cu^2+^ ions concentration, where I and I_0_ are the photocurrent intensities of CdS-rGO in the presence and absence of Cu^2+^ ions, respectively. The inset of **Fig 6(B)** shows an excellent linear relationship of R^2^ = 0.9915. The regression equation was ΔI (μA) = 13.89 + 6.97 log [Cu^2+^] (μM) between the value of ΔI and the logarithm of the Cu^2+^ ions concentration of 0.5–120.0 μM. The limit of detection (LoD) was evaluated using 3σ/S and was found to be 0.016 μM, where σ is the standard deviation of a blank signal, and S is the slope of the linear calibration plot. A possible schematic mechanism for the PEC discernment of Cu^2+^ ions is shown in **Fig 7**. Under illumination, the photogenerated electrons are transferred from the valence band (VB) to the conduction band (CB) of a CdS nanoparticles present in the CdS-rGO nanocomposite, and then holes are generated in the VB. The photogenerated holes are scavenged by TEA, and the electrons are promoted to the CB of CdS. Hence, when CdS was in contact with graphene, the photogenerated electrons in the CB of the CdS were transferred to the two-dimensional carbon sheets, which hindered the charge recombination and prolonged the lifetime of the photogenerated carriers. The photocurrent intensity was greatly decreased upon the addition of Cu^2+^ ions. The addition of Cu^2+^ ions to CdS resulted in the binding of Cu^2+^ with S^2-^, and the reduction of Cu^2+^ to Cu^+^ ions under irradiation. Consequently, Cu_x_S (x = 1, 2) was formed on the surface of CdS because Cu^2+^ chemically displaced Cd^2+^ because Cu_x_S has a lower solubility than CdS. The development of recombination centers (Cu_x_S) on the CdS surface generated a low band gap energy, which effectively recombined excited electrons in the CB and holes in the VB [49–52]. In addition, to prove the quenching of the photocurrent upon the addition of Cu^2+^ ions, FESEM images were recorded for the CdS-rGO nanocomposite after the addition of Cu^2+^ ions to the photoelectrochemical cell. In **Fig 6(C)**, a small hole is observed in some of the spherical CdS structures due to the presence of 1.15% Cu content in the CdS-rGO nanocomposite. The transparent ultrathin rGO sheets were seen to retain their role of linking the spherical CdS, even after the addition of 4 μM Cu^2+^ ions under an illuminated condition. A comparison of this FESEM image with **Fig 6(B)** clearly indicates a photocurrent quenching due to the formation of Cu_x_S, *i*.*e*., analyte-induced morphological changes caused by photocurrent quenching. The presence of CdS in the composite was demonstrated by the Cd and S peaks in the EDX profile (**Fig 6(D)**). The spectrum reveals an atomic Cd to S ratio of about 1:1, which agrees with the stoichiometric CdS. The C came from graphene, and the O was contributed by the residual oxygenous groups on the rGO. Thus, the result indicates that the CdS was successfully deposited on the rGO sheets. In contrast, the small peak observed in the EDX spectrum implies the existence of 0.55 atomic % of Cu^2+^ (**Fig 6(D), inset**). Therefore, the photocurrent intensity of the CdS-rGO decreased in the Cu^2+^ ion solution, making the nanocomposite suitable for the selective determination of Cu^2+^ ions.

Even though much research has been reported on the PEC sensing of Cu^2+^ ions, our current work demonstrated a facile, environmentally friendly, and low-cost method, especially in relation to the synthesis of a modified electrode. There has been no work reported based on the AACVD synthesis of CdS using cadmium acetate and thiourea as the precursors. In addition, an ITO/CdS-rGO electrode holds the promise of high sensitivity toward Cu^2+^ ions, without requiring a ternary hybrid such as Cd_x_Zn_1-x_S–rGO [46] and MPA-capped CdTe/CdS [53]. Some of the Cu^2+^ ions sensors with various detection methods are presented in **Table 1**. As shown in **Table 1**, the LoD obtained from the ITO/CdS-rGO-modified electrode was the lowest compared to the other reported works. Our LoD of 0.016 μM is far below the WHO’s guideline value of ~20 μM that is allowed for Cu^2+^ ions in drinking water. This indicates that the proposed PEC sensor is ultra-sensitive and holds promise for the detection of a trace amount of Cu^2+^ ions in drinking water.

**Table 1:** Comparison of various Cu^2+^ ions sensor based on various detection methods.

Detection method	Electrode/substrate	Electrode preparation	Linear range (μM)	LoD (μM)	Ref.
Photoelectrochemical sensor	Cd_x_Zn_1-x_S–rGO on GCE	facile one-pot reaction	0.02–20	0.067	[46]
Photoelectrochemical sensor	SnO_2_/CdS heterostructural films on FTO	SILAR	1.0–38.0	0.55	[11]
Electrochemiluminescence	MPA-capped CdTe/CdS	In-situ	0.1–10	0.02	[53]
Luminescent chemosensor	CdS cluster, [Cd_10_S_4_(SR)_12_]	Ligand-exchange reaction	10	0.07	[54]
Luminescent response	L-cysteine and thioglycerol-capped CdS QD	Reflux	1600	1	[50]
Fluorescence resonance energy transfer (FRET)	Phenol formaldehyde resin PFR-CdTe QDs	Hydrothermal	0.80–5.36	0.16	[55]
Adsorption stripping voltametry (AdSV)	CPE modified with Ac-Phos SAMMS	Adsorption of monolayers	0.160–3.147	0.0786	[56]
Potentiometric method using ion-selective electrodes	CPE modified with MWCNT	In-situ	0.01–10000	1.07	[57]
Differential pulse voltammetry measurement	Carbon tip electrode modified with mercury film	Electrochemical deposition	9.44–157.37	1.57	[58]
Photoelectrochemical sensor	CdS-rGO on ITO	AACVD	0.5–120	0.016	Present work

### 3.7. Interference studies

**Fig 8:**
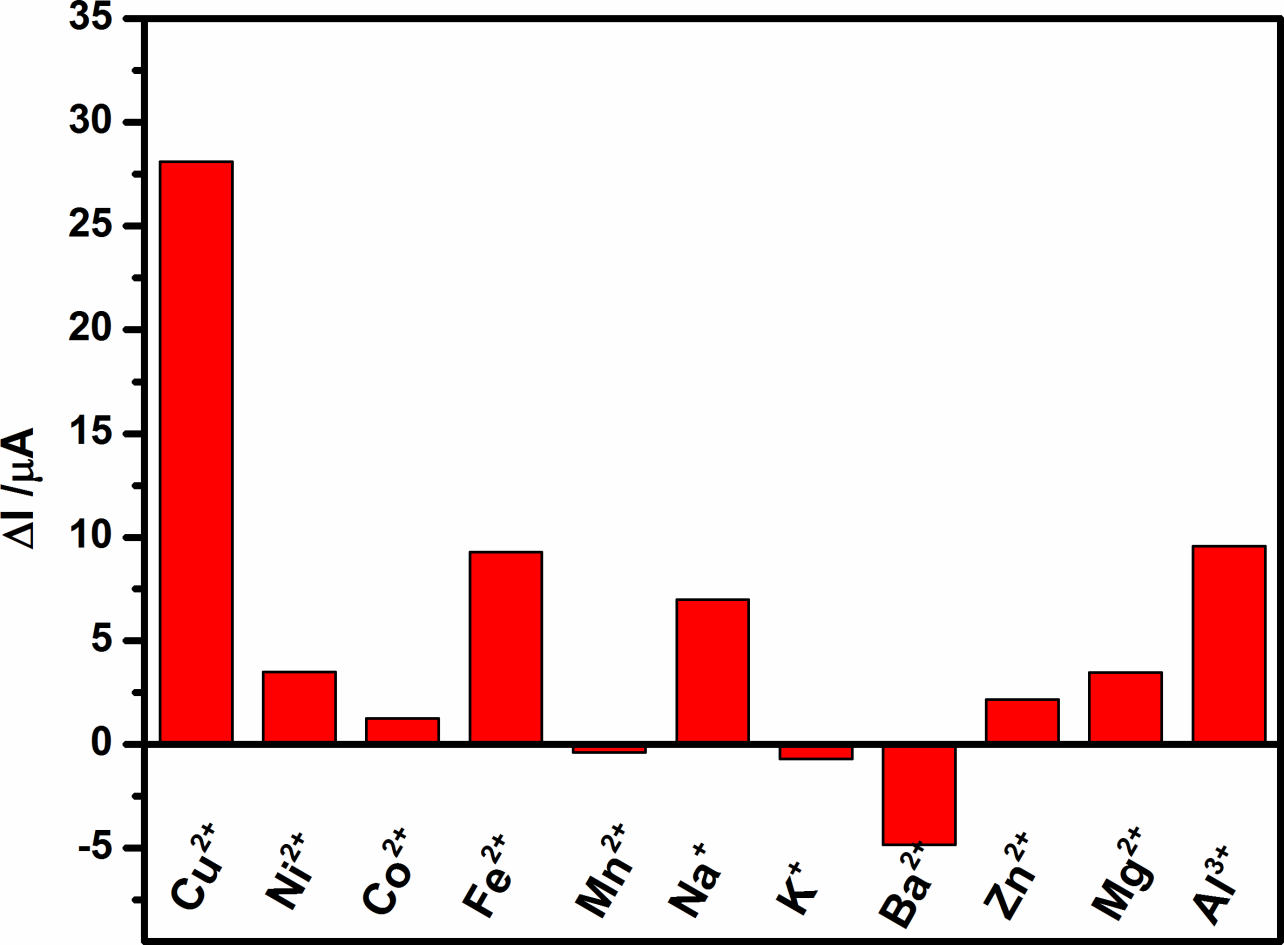
Photocurrent changes in prepared ITO/CdS-rGO electrode toward 0.5 μM Cu^2+^ ions against 120 μM of other metal ions.

The selectivity of a Cu^2+^ ions assay was determined by investigating the influences of various kinds of metal ions at the same concentration of 120 μM, including Ni^2+^, Co^2+^, Fe^3+^, Mn^2+^, Na^+^, K^+^, Ba^2+^, Zn^2+^, Mg^2+^, and Al^3+^, on the photocurrent response of the prepared ITO/CdS-rGO electrode when the concentration of Cu^2+^ ions was at 0.5 μM. As shown in **Fig 8**, even with a 240-fold excess of another metal ion (120 μM vs. 0.5 μM Cu^2+^ ions), no significant photocurrent change can be seen in comparison with Cu^2+^ ions. In addition, it was observed that the photocurrent intensity of the ITO/CdS-rGO electrode was also decreased by Ni^2+^, Co^2+^, Fe^3+^, Na^+^, Zn^2+^, Mg^2+^, and Al^3+^. This was due to the displacement of Cd^2+^, leading to the formation of MS (for M = Ni, Co, Fe, Na, Zn, Mg, Al) on the CdS surface [31]. The formed MS behaved as sites for recombining photogenerated electron–hole pairs due to its defects on the surface and lower band energy. However, the ITO/CdS-rGO electrode was significantly sensitive to Cu^2+^ ions compared to the other metal ions because CuS has a notably lower K_sp_ value than NiS, CoS, FeS, and ZnS [31]. Hence, the prepared ITO/CdS-rGO electrode was sensitive and selective toward Cu^2+^ ions.

## Conclusion

A PEC sensor based on the CdS-rGO nanocomposite was successfully fabricated using AACVD and dip-coating strategies. The CdS-rGO nanocomposite showed the highest PEC activity compared to that of bare GO, rGO, CdS, and CdS-GO electrodes under visible-light illumination. This was because the presence of rGO facilitated the transfer of electrons from the conduction band of the CdS, thus manifesting the photocurrent performance of the nanocomposite. The photocurrent intensity of the CdS-rGO nanocomposite was effectively suppressed by the displacement of Cd^2+^ by Cu^2+^. This phenomenon resulted in a PEC sensor with heightened sensitivity and selectivity toward Cu^2+^ ions, manifesting its potential in real applications.

## Supporting Information

S1 Fig LSV obtained for ITO/CdS-rGO photoelectrode dipped into (A) 0.1 M KCl, (B) mixture of 0.1 M KCl and 0.5 M TEA, and (C) 0.1 M KCl, 0.5 M TEA, and 4 μM Cu (II) under (a) light irradiation, (b) dark condition, and (c) light “on-off” condition. (D) LSV responses in (a) absence and (b) presence of 4 μM Cu^2+^ ions with 0.1 M KCl and 0.5 M TEA under light “on-off” condition at scan rate of 0.1 Vs^–1^.
(DOCX)
