# Supplementary material for: Cadmium Sulphide-Reduced Graphene Oxide-Modified Photoelectrode-Based Photoelectrochemical Sensing Platform for Copper(II) Ions
Source: PLoS One. 2016 May 13;11(5):e0154557. doi: 10.1371/journal.pone.0154557 (PMC4866701; doi:10.1371/journal.pone.0154557)
Supplement: S1 Fig — LSV obtained for ITO/CdS-rGO photoelectrode dipped into (A) 0.1 M KCl, (B) mixture of 0.1 M KCl and 0.5 M TEA, and (C) 0.1 M KCl, 0.5 M TEA, and 4 μM Cu (II) under (a) light irradiation, (b) dark condition, and (c) light “on-off” condition. (D) LSV responses in (a) absence and (b) presence of 4 μM Cu2+ ions with 0.1 M KCl and 0.5 M TEA under light “on-off” condition at scan rate of 0.1 Vs–1. (DOCX) [file pone.0154557.s001.docx]

**Cadmium Sulphide-Reduced Graphene Oxide-Modified Photoelectrode-Based Photoelectrochemical Sensing Platform for Copper(II) Ions**

**I. Ibrahim^1^, H.N. Lim^1,2*^, N.M. Huang^3^, A. Pandikumar^3**^**

^1^Department of Chemistry, Faculty of Science, Universiti Putra Malaysia, 43400 UPM Serdang, Selangor, Malaysia

^2^Functional Device Laboratory, Institute of Advanced Technology, Universiti Putra Malaysia, 43400 UPM Serdang, Selangor, Malaysia

^3^Low Dimensional Materials Research Centre, Department of Physics, Faculty of Science, University of Malaya, Kuala Lumpur 50603, Malaysia

*Corresponding author: [hongngee@upm.edu.my](mailto:hongngee@upm.edu.my) (Hong Ngee Lim)

**Corresponding author: [pandikumarinbox@gmail.com](mailto:pandikumarinbox@gmail.com) (Alagarsamy Pandikumar)


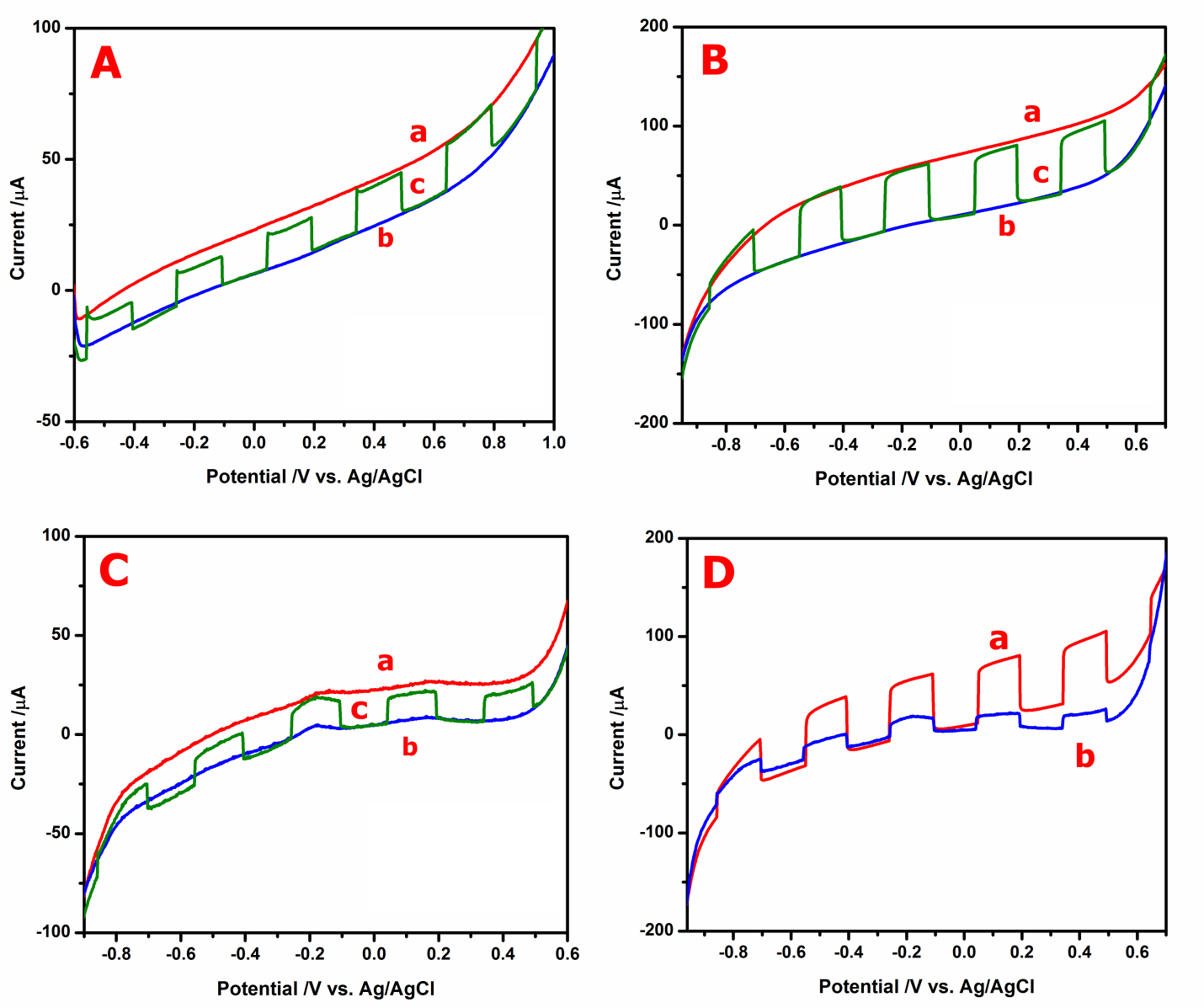


**S1 Fig.**  LSV obtained for ITO/CdS-rGO photoelectrode dipped into (A) 0.1 M KCl, (B) mixture of 0.1 M KCl and 0.5 M TEA, and (C) 0.1 M KCl, 0.5 M TEA, and 4 µM Cu (II) under (a) light irradiation, (b) dark condition, and (c) light “on-off” condition. (D) LSV responses in (a) absence and (b) presence of 4 µM Cu^2+^ ions with 0.1 M KCl and 0.5 M TEA under light “on-off” condition at scan rate of 0.1 Vs^–1^.
